# Draft Genome Sequence of *Pseudomonas aeruginosa* ATCC 9027, Originally Isolated from an Outer Ear Infection

**DOI:** 10.1128/genomeA.01397-17

**Published:** 2017-11-30

**Authors:** Ambikesh Jayal, Benjamin E. Johns, Kevin J. Purdy, Sarah E. Maddocks

**Affiliations:** a Department of Computing and Information Systems, School of Management, Cardiff Metropolitan University, Cardiff, United Kingdom; b Department of Biomedical Sciences, Cardiff School of Sport and Health Sciences, Cardiff Metropolitan University, Cardiff, United Kingdom; c School of Life Sciences, University of Warwick, Coventry, United Kingdom

## Abstract

*Pseudomonas aeruginosa* ATCC 9027 was isolated in 1943 from a case of otitis externa and is commonly employed as a quality control strain for sterility, assessment of antibiofilm agents, and *in vitro* study of wound infection. Here, we present the 6.34-Mb draft genome sequence and highlight some pertinent genes that are associated with virulence.

## GENOME ANNOUNCEMENT

*Pseudomonas aeruginosa* ATCC 9027 was originally isolated from a case of otitis externa and is used as a quality control organism (https://www.lgcstandards-atcc.org/Products/Cells_and_Microorganisms/Bacteria.aspx). Despite its origin as a skin pathogen, this strain has been regarded as avirulent and has been utilized to produce rhamnolipid surfactants (1, 2). Nonetheless, *P. aeruginosa* ATCC 9027 has been used extensively to study wound infection *in vitro*, because it is an excellent biofilm former (3). *P. aeruginosa* ATCC 9027 is closely related to PA7, a taxonomic outlier which exhibits numerous multidrug resistance mechanisms (4). PA7 lacks several factors associated with virulence, including a 36-gene cluster encoding the type III secretory system (5).

Genomic DNA derived from a biofilm of *P. aeruginosa* ATCC 9027 was purified using the GenElute bacterial genomic DNA extraction kit (Sigma-Aldrich). The sample library was prepared using an Illumina Nextera XT before DNA sequencing using an Illumina MiSeq system. The genomic data were transferred to and stored in Illumina BaseSpace. The raw reads were processed for quality using Trimmomatic version 0.36 (6) and quality reports generated using FastQC (7). A 6,340,907-bp assembly with 126-fold coverage was constructed using SPAdes version 3.8 (8) and run remotely using Cloud Infrastructure for Microbial Bioinformatics (CLIMB) (9, 10). Quality assessment of assembly was done using QUAST (11). The assembly consists of 53 contigs (500 bp or more) with a mean G+C content of 66.65%. The *N*_50_ and *N*_75_ values of the assembly were 290,774 bp and 197,962 bp, respectively. The *L*_50_ and *L*_75_ values of the assembly were 8 and 15, respectively. The annotation was performed with the NCBI Prokaryotic Genome Annotation Pipeline (http://www.ncbi.nlm.nih.gov/genome/annotation_prok), which predicted a total of 5,841 protein-coding genes and 58 tRNAs.

The draft genome contained several virulence-associated genes, including *flg*, *pil*, and *gld* (flagellar and fimbrial biosynthesis and motility [12, 13]), *mviM* (putative virulence factor [http://www.uniprot.org/uniprot/Q1RD89]), *hudR* (virulence-associated transcriptional regulator [14]), the *pvd* operon (pyoverdin synthesis [15]), *bvgS* (virulence sensor protein [16]), *mvfR-pqsR* (multiple virulence factor regulator [17]), *vreR* (sigma factor regulator associated with virulence [18]), and *hemO* (heme oxygenase [19]), to name a few. The presence of these genes demonstrates that *P. aeruginosa* ATCC 9027 should be classified as pathogenic, rather than avirulent, and could have implications for its use as a workhorse to produce surfactants.

### Accession number(s).

This whole-genome shotgun project has been deposited at DDBJ/ENA/GenBank under the accession number PDLX00000000. The version described in this paper is version PDLX01000000. Unassembled reads are also available from the NCBI Sequence Read Archive (SRA) under the accession number SRP120030.
